# Acupuncture for Psychosomatic Symptoms of Hwa-byung, an Anger Syndrome: A Feasibility Randomized Controlled Trial

**DOI:** 10.3389/fpsyg.2021.651649

**Published:** 2021-09-24

**Authors:** Yujin Choi, In-Hye Park, Jung-Eun Kim, Ojin Kwon, Ae-Ran Kim, Hyo-Ju Park, Jun-Hwan Lee, Joo-Hee Kim

**Affiliations:** ^1^KM Science Research Division, Korea Institute of Oriental Medicine, Daejeon, South Korea; ^2^Department of Korean Medicine, College of Korean Medicine, Sangji University, Wonju, South Korea; ^3^Biomedical Research Institute, Pusan National University Hospital, Busan, South Korea; ^4^R&D Strategy Division, Korea Institute of Oriental Medicine, Daejeon, South Korea; ^5^Korean Medicine Life Science, University of Science & Technology (UST), Campus of Korea Institute of Oriental Medicine, Daejeon, South Korea; ^6^Department of Acupuncture and Moxibustion Medicine, College of Korean Medicine, Sangji University, Wonju, South Korea; ^7^Research Institute of Korean Medicine, Sangji University, Wonju, South Korea

**Keywords:** acupuncture, anger syndrome, Hwa-byung, stuffiness, psychosomatic symptoms, randomized controlled trial, feasibility trial

## Abstract

**Objectives:** Emerging studies found the potential effects of acupuncture for treating chronic pain and mental disorders, namely, depressive and anxiety disorders. Acupuncture is widely used for treating culture-related anger syndrome, Hwa-byung. This pilot trial aimed to investigate the feasibility of a clinical trial testing acupuncture for the psychosomatic symptoms of Hwa-byung.

**Methods:** A total of 26 patients with Hwa-byung planned to be randomly assigned to the acupuncture or sham acupuncture groups. About 10 treatment sessions were applied over 4 weeks. The 100-mm visual analog scale was used to measure the six major Hwa-byung symptoms: stuffiness in the chest, heat sensations, pushing-up in the chest, feeling a mass in the throat, feelings of unfairness, and hard feelings. The criteria for assessing the success of this pilot trial were defined as improvement in three or more of the six Hwa-byung symptoms after treatment, with an effect size >0.2.

**Results:** A total of 15 patients were finally included and randomly assigned to the acupuncture group (*n* = 7) or the sham acupuncture group (*n* = 8). After 10 treatment sessions, the Cohen's *d* effect sizes for acupuncture compared to sham acupuncture were >0.2 for each one of the six major Hwa-byung symptoms, which met our *a priori* criteria for success. Also, the effect size for the somatic symptoms of “stuffiness in the chest” was 0.81 (95% CI −0.40, 2.20), referring to a large effect size.

**Conclusions:** Our results suggest that acupuncture treatment would be regarded as an acceptable intervention for a full-scale study of psychosomatic symptoms in patients with Hwa-byung.

**Trial Registration:** cris.nih.go.kr, identifier: KCT0001732.

## Introduction

Hwa-byung, a culture-bound syndrome in Korea, can be understood as a type of somatoform disorder, having typical somatic symptoms of stuffiness in the chest (Lee, 1997; Kwon et al., 2020). Hwa-byung is likely to develop in people with anger suppressed for long durations (Lin, 1983). The representative symptoms of Hwa-byung include stuffiness in the chest, heat sensations, pushing-up in the chest, feeling a mass in the throat, feelings of unfairness, and hard feelings (Min et al., 2009), which are also mainly included in diagnostic criteria of Hwa-byung (The Korean Society of Oriental Neuropsychiatry Hwabyung Research Center, 2013). An epidemiological study found 4.1–13.3% of the general population of Korea to be suffering from Hwa-byung, and the syndrome is more widely prevalent in middle-aged women than in men (Nam et al., 1990). The most common factor of Hwa-byung was reported to be conflict within the family relationship, which is one of the most highly regarded values in Korean culture (Suh, 2013; Lee et al., 2014).

Hwa-byung is not only confined to Korean society, since anger is one of the most universal and basic human emotions that can be found under different names in other cultures, namely, “trapped housewife syndrome” in the USA (Min, 2008) and “Ataques De Nervios” in Mexico. Also, Hwa-byung is a distinctive disease that is differentiated from other psychiatric disorders. It is suggested that Hwa-byung is more likely to be a psychiatric syndrome mainly suffering from somatic symptoms, rather than a variant of depression or anxiety disorder (Lee et al., 2012b).

Acupuncture is a universal and safe therapeutic method for various diseases, namely, psychiatric disorders. Acupuncture is known to be effective for chronic pain (Vincent, 1990; Vickers et al., 2012). Also, potential effects of acupuncture for relieving depression and anxiety have been tested in various clinical studies (Macpherson et al., 2013; Smith et al., 2018; Li et al., 2019), although the low quality of supporting evidence is still challenging. In clinical practice, acupuncture is also widely used for treating individuals with Hwa-byung (Jung et al., 2008; Min and Suh, 2010; Choi et al., 2011; Lee et al., 2012a). Acupuncture has the potential for stress reduction (Wild et al., 2020) and relieving sympathetic activation (Middlekauff et al., 2002). It may alleviate the various psychosomatic symptoms of patients with Hwa-byung by reducing the stress response.

To date, five small-size randomized clinical trials of acupuncture for the patient with Hwa-byung have been reported, and the effect of acupuncture for Hwa-byung symptoms is unclear. In previous studies, mainly *Saam* acupuncture using four acupoints was used in four clinical trials for relieving Hwa-byung symptoms (Lee et al., 2007; Jung et al., 2008; Choi et al., 2011, 2015), and scalp acupuncture was used in one trial for relieving insomnia in patients with Hwa-byung (Lee et al., 2012a). In clinical practice, manual acupuncture using 10–20 acupoints is commonly used (Lee et al., 2009), but the effect of individualized manual acupuncture treatment for Hwa-byung has not been tested. Also, there is a lack of evidence of acupuncture relieving the psychosomatic symptoms of Hwa-byung, namely, stuffiness in the chest.

In this feasibility trial, we aimed to explore the efficacy and safety of semiindividualized acupuncture treatment as a method to reflect the optimal effect of acupuncture for anger-related psychosomatic symptoms of patients with Hwa-byung in real clinical practice. The objectives of this trial to assess the feasibility and to obtain basic information for further large-scale research were as follows.

1. To evaluate the acceptability of acupuncture in potential efficacy and safety for a definitive trial;
2. To explore candidates of the primary outcome and calculate sample size for a definitive trial; and
3. To investigate the feasibility of the study design, namely, recruitment, compliance, and completion rate for a definitive trial.

## Methods

### Trial Design

We conducted a randomized, parallel-group, sham-controlled, and participant- and assessor-blinded clinical trial. Participants were randomly assigned to the acupuncture or sham acupuncture groups in a 1:1 ratio. The protocol was approved by the Institutional Review Board of the Daejeon Oriental Hospital of Daejeon University (approval no. djomc-133). This trial was prospectively registered at the Clinical Research Information Service (KCT0001732).

### Participants

The inclusion criteria were men and women aged from 20 to 65 years who met the diagnostic criteria of Hwa-byung. The main exclusion criteria were history of serious psychiatric or neurologic disorder, use of medications related to Hwa-byung such as antidepressants during preceding 1 month, seriously unstable medical condition. The detailed inclusion and exclusion criteria can be found in a published protocol (Lee et al., 2018). This pilot trial was conducted at the outpatient department of a university hospital in Daejeon. The eligible participants were recruited through the participant recruitment poster displayed at the site and newspaper advertisements. The participants who voluntarily applied to the trial were informed about the study in detail from a Korean medicine doctor, and written consents for participation were obtained before the screening process.

### Interventions

Stimulation on the classical acupoints was applied in the acupuncture group, while minimal acupuncture stimulation of non-classical acupoints was applied in the sham acupuncture group. During the 4-week treatment period, 10 sessions of intervention were applied, two to three times per week. In both groups, 0.25 × 30 mm disposable and sterile stainless steel acupuncture needles (Dongbang, Republic of Korea) were used.

In the acupuncture group, semiindividualized acupoints were used, which included the fixed acupoints of GV20 (Kim et al., 2012) on the head, CV17 (Cho et al., 2009) on the chest, HT7 on the wrist bilaterally, ST36 on the legs bilaterally, and two individualized acupoints. The two individualized acupoints were previously planned according to the pattern identification (Kim et al., 2013): LR3 and PC6 for the stagnation of liver qi, which refers to an excess pattern of Hwa-byung; and HT5 and KI3 for disharmony between the heart and the kidney, which refers to the deficiency pattern of Hwa-byung. Acupuncture was applied with a depth of 5.0–25.0 mm, and for the acupuncture response, the De-qi sensation was obtained.

In the sham acupuncture group, shallow needle insertion was applied to a depth of 1.0–3.0 mm with no manipulation for *De qi*, on nonclassical acupoints. Ten points (bilateral) on the upper arms, abdomen, and legs were selected, which were not located in the classical meridian: upper extremities 1, 1 cm lateral from 5 cm below the midpoint of the transverse cubital crease; abdomen 1, 3 cm lateral from a point 13.5 cm above the umbilicus; lower extremities (LE) 1, 1.5 cm above EX-LE 2; LE 2, the point at the upper one-third of the medial part of the tibia; and LE 3, 1.5 cm below LE 2.

### Randomization and Blinding

Random sequence numbers were generated using SAS^®^ version 9.4 (SAS Institute Inc., Cary, NC, USA) by an independent statistician. The group assignments were kept secured within opaque envelopes, and the envelopes were opened right after the participant enrollment for the allocation concealment. The practitioners could not be blinded, as they performed the acupuncture manipulations and sham acupuncture. This study was designed for the blinding of the participants and assessors. The success of blinding was tested at the end of the treatment period by the method of Bang's new blinding index (Bang et al., 2004). Participants were informed that they would be treated with either classical acupuncture or non-classical acupuncture. At the end of the treatment, they were asked to choose which treatment was applied to them, including the answer of “do not know.” Bang's new blinding index ranges from −1 to 1. The closer the index is to 1, it means that the more people correctly answered the treatment they actually received. On the other hand, the closer the index is to −1, it means that the more people answered the opposite treatment they actually received.

### Outcome Measurement and Feasibility Criteria

The acceptability of acupuncture for Hwa-byung symptoms to conduct a definitive trial was evaluated in potential efficacy and safety. The criteria for assessing the success of the pilot trial were previously defined (Lee et al., 2018). Concerning efficacy, the Cohen's *d* effect sizes of >0.2 in at least three of the six major Hwa-byung symptoms at week 4 (posttreatment) compared to control, would indicate acupuncture treatment as an acceptable intervention for a large-scale clinical trial for Hwa-byung. Six major Hwa-byung symptoms were extracted from the diagnostic criteria of Hwa-byung (The Korean Society of Oriental Neuropsychiatry Hwabyung Research Center, 2013; Kwon et al., 2020). There are four major somatic symptoms: stuffiness in the chest, heat sensations, pushing-up in the chest, and feeling a mass in the throat. Also, there are two major psychological symptoms; feelings of unfairness and hard feelings or “*Haan*,” which refers to mixed feelings of missing someone, sorrow, regret, sadness, and depression, along with some feelings of hatred and revenge. A 100-mm visual analog scale (VAS) was used to measure the severity of the six major Hwa-byung symptoms. Concerning safety, in case of no significant difference in the number of intervention-related adverse events observed between the two groups and no intervention-related serious adverse events occurred in this pilot trial, a further trial would be planned. During the entire study period, the occurrence of adverse events was recorded at every visit, and the severity and causality of adverse events were assessed.

To explore the candidates of the primary outcome for further trial, the Beck Depression Inventory-II (BDI-II) (Beck et al., 1996; Lim et al., 2011) and the short form of the Stress Response Inventory [SRI-short form (SRI-SF)] (Choi et al., 2006) were also measured. BDI-II is a representative self-rating scale for depression and ranges from 0 to 63. SRI-SF includes three subscales of somatization, depression, and anger. The somatization, depression, and anger subscale of SRI-SF consists of 9, 8, and 5 items, and ranges from 0 to 36, 0 to 32, and 0 to 20. SRI-SF was used to evaluate stress response in patients with Hwa-byung. The clinical outcomes were assessed at week 0 (baseline), week 2 (undertreatment), week 4 (posttreatment), and week 8 (follow-up).

The recruitment rate per week, compliance of participants, and completion rate were observed to determine the number of sites and feasible recruitment plan for further trial.

### Statistical Analyses

The numbers in this pilot trial were set at 26 participants, referring to the minimum number required to assure the validity of mean, SD, and effect size (Julious, 2005). It was not determined based on power calculation for hypothesis testing.

To evaluate the acceptability of acupuncture, the Cohen's *d* effect size was calculated as the difference between mean changes from baseline at week 4 (posttreatment) of the two groups divided by the estimated standardized deviation (Cohen, 1992). The 95% CIs of Cohen's *d* were obtained by bootstrap using the R package rstatix (Kassambara, 2020). Also, the proportion of the number of intervention-related adverse events by the total number of interventions applied in the two groups was compared by the chi-squared test or Fisher's exact test.

To explore the clinical outcomes measured at week 0, week 2, week 4, and week 8, repeated measures ANOVA were carried out. Times were used as within-subjects factors, and the groups were used as between-subjects factors. As the *post-hoc* tests, simple main effects of the group at each time point were investigated, and pairwise comparisons between the groups at each time point were carried out. The *p*-values were adjusted by the Bonferroni method. Also, the adjusted mean and SD of the two groups and mean difference (95% CI) at each time point were calculated by the analysis of covariance, using the baseline values as covariates and the group as the fixed factor. The analysis was performed using SAS^®^ version 9.4 (SAS Institute Inc., Cary, NC, USA). There was no missing data and no need for imputation. All subjects were treated with an overall compliance of more than 80%, and there were no major protocol violations.

To estimate the recruitment, compliance, and completion rate, Wilson 95% CIs for binomial probabilities were calculated (Brown et al., 2001).

## Results

### Participant Flow and Baseline Characteristics

**Figure 1:**
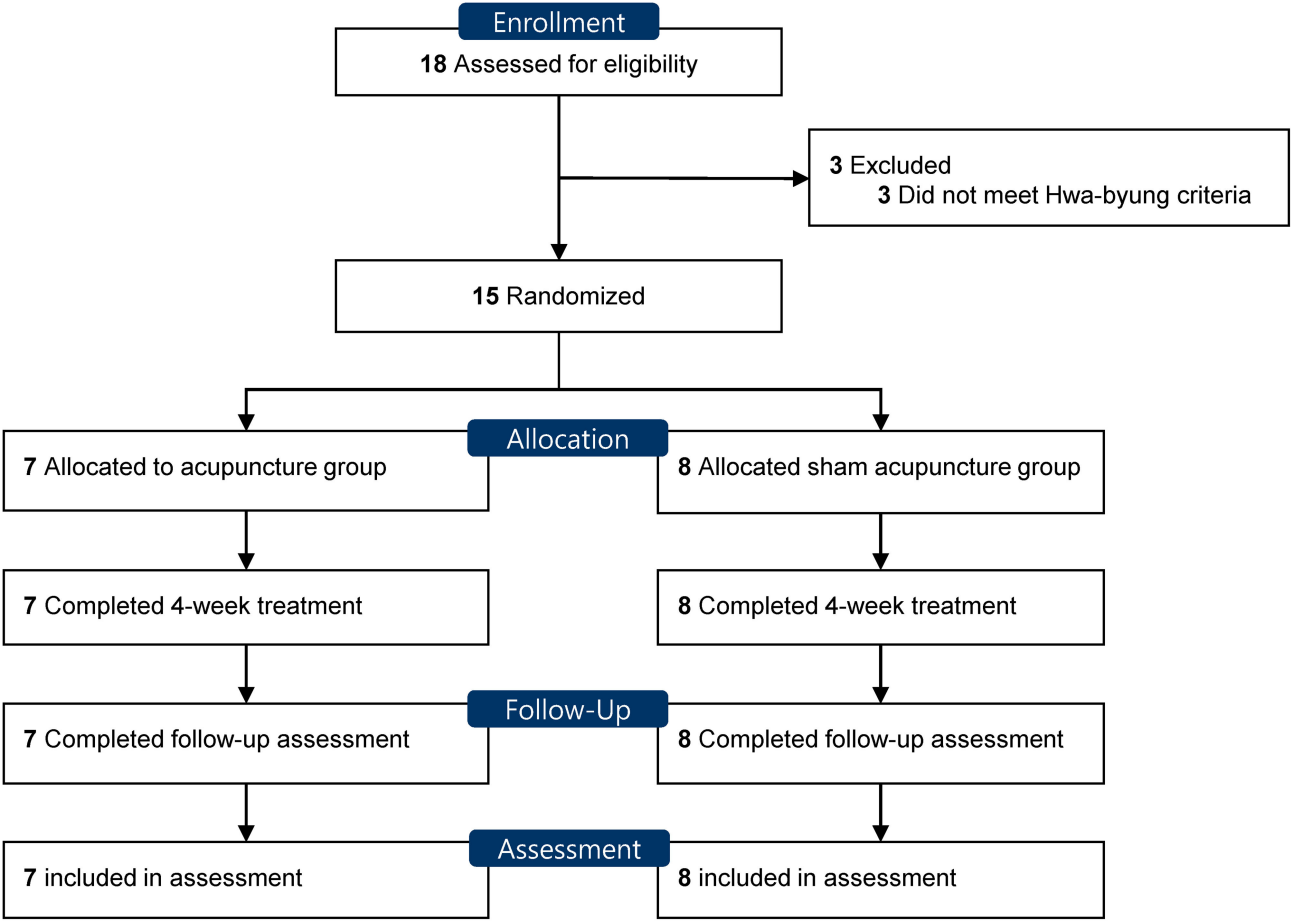
Flow diagram of participants in the feasibility trial.

Figure 1 shows the flow of participants. From January 2016 to December 2016, 18 participants were screened, and 15 participants were included. Three patients who failed to meet the diagnostic criteria of Hwa-byung were excluded. The 15 eligible participants were randomly allocated to the acupuncture group (*n* = 7) and the sham acupuncture group (*n* = 8). No baseline demographic or characteristic showed a significant difference between the groups (Table 1). The pattern of the Hwa-byung was also distributed in balance between the two groups, and the liver-qi stagnation pattern was the most frequent pattern in both groups, followed by non-interaction between the heart and the kidney patterns. This pilot trial ended before recruiting a planned number of 26, due to budget and time limit, after recruiting participants for 1 year.

**Table 1:** Participant baseline characteristics.

**Characteristics**	**Acupuncture group (*n* = 7)**	**Sham acupuncture group (*n* = 8)**	***p*-value**
Sex			0.4667
Male	1 (14.29%)	0 (0.00%)	
Female	6 (85.71%)	8 (100.00%)	
Age (year)	53.86 (4.71)	52.13 (12.84)	0.7420
Education (year)			0.1026
6~8 years	1 (14.29%)	0 (0.0%)	
9~11 years	0 (0.00%)	1 (12.50%)	
12~15 years	1 (14.29%)	5 (62.50%)	
≥ 16 years	5 (71.43%)	2 (25.00%)	
Duration of Hwa-byung symptoms (month)	82.29 (100.39)	85.50 (99.14)	0.9403
Pattern of Hwa-byung			0.9999
Liver-qi stagnation	4 (57.14%)	5 (62.50%)	
Non-interaction between the heart and kidney	3 (42.86%)	3 (37.50%)	
**Major Hwa-byung symptoms (VAS, 0–100 mm)**			
Stuffiness in the chest	83.43 (18.73)	72.25 (12.63)	0.2100
Heat-sensation	67.57 (35.76)	66.88 (20.48)	0.9650
Pushing-up in the chest	87.29 (17.42)	69.62 (33.44)	0.2190
Feeling a mass in the throat	80.57 (30.42)	61.25 (27.65)	0.2240
Feeling of unfairness^a^	97.00 (15.50)	89.00 (15.75)	0.2950
Hard feelings or *Hann*^a^	97.00 (21.50)	93.50 (14.75)	0.9530
BDI-II (0–63)	48.71 (6.32)	49.12 (7.74)	0.9120
**Short form of SRI**			
Somatization (0–36)	22.14 (9.72)	20.75 (5.09)	0.7420
Depression (0–32)	18.43 (8.92)	22.00 (6.48)	0.4000
Anger (0–20)	15.43 (3.95)	11.25 (5.70)	0.1210

*The data are shown as mean (SD), median (interquartile range), or frequency (%)*.

*VAS, visual analog scale; BDI-II, Beck Depression Inventory-II; SRI, Stress Response Inventory*.

a *Variables which are not normally distributed. Data are shown as median (interquartile range), and p-values were calculated by Wilcoxon tests*.

### Acceptability of Acupuncture in Potential Effect

The criteria for assessing the success of this pilot trial were defined as three or more major symptoms of Hwa-byung to be improved after the 4-week acupuncture treatment compared to sham, with an effect size >0.2. The effect sizes of acupuncture for the major Hwa-byung symptoms at week 4 (posttreatment) are presented in Table 2. Every VAS of the six Hwa-byung symptoms showed the Cohen's *d* effect size >0.2. The Cohen's *d* effect size of acupuncture for VAS of “stuffiness in the chest” was 0.81 (95% CI −0.40, 2.20), which was >0.8, referring to the large effect size. Symptoms of “pushing-up in the chest,” “feeling mass in throat,” and “feeling of unfairness” showed Cohen's *d* >0.5, which means moderate effect size (Cohen, 1992).

**Table 2:** The effect size of acupuncture on major Hwa-byung symptoms measured on a 100-mm visual analog scale, BDI-II, and SRI-short form at week 4 (posttreatment).

**Change from baseline at week 4**	**Acupuncture**	**Sham acupuncture**	**Mean difference (95% CI)**	**Cohen's *d* (95% CI)**	**Effect size magnitude**
**Major Hwa-byung symptoms**					
Stuffiness in the chest	−44.29 (35.96)	−21.00 (19.41)	−23.29 (−57.75, 11.18)	0.81 (−0.40, 2.20)	Large
Heat–sensation	−32.57 (51.65)	−18.50 (25.26)	−14.07 (−63.11, 34.97)	0.35 (−1.37, 1.70)	Small
Pushing–up in the chest	−46.14 (43.42)	−14.88 (36.73)	−31.27 (−76.92, 14.38)	0.78 (−0.26, 2.21)	Moderate
Feeling a mass in the throat	−42.71 (45.09)	−23.62 (24.60)	−19.09 (−62.37, 24.19)	0.53 (−0.70, 2.10)	Moderate
Feeling of unfairness	−49.29 (38.77)	−31.50 (26.39)	−17.79 (−56.30, 20.73)	0.54 (−0.65, 2.12)	Moderate
Hard feelings or *Han*^a^	−41.29 (45.05)	−31.88 (27.70)	−9.41 (−53.35, 34.53)	0.25 (−1.20, 1.70)	Small
**BDI–II**					
BDI–II	−10.14 (7.54)	−7.12 (8.97)	−3.02 (−12.23, 6.19)	0.36 (−0.79, 1.48)	Small
**Short form of SRI**					
Somatization	−12.71 (7.54)	−4.25 (3.15)	−8.46 (−15.55, −1.38)	1.46 (0.32, 5.50)	Large
Depression	−8.86 (9.72)	−7.75 (7.61)	−1.11 (−11.09, 8.88)	0.13 (−0.97, 1.28)	Negligible
Anger	−6.29 (4.68)	−2.75 (3.54)	−3.54 (−8.30, 1.23)	0.85 (−0.19, 2.26)	Large

*Data are shown as mean (SD) or estimate (95% CI)*.

*BDI–II, Beck Depression Inventory-II; SRI, Stress Response Inventory*.

a *Mixed feelings of missing someone, sorrow, regret, sadness, and depression, along with some feelings of hatred*.

### Acceptability of Acupuncture in Potential Safety

Concerning the criteria of safety, no severe adverse events occurred throughout the study in either group (Supplementary Table 1). Five adverse events were reported in each group. Of the total 10 adverse events reported, only one adverse event (fatigue in the acupuncture group) was determined to be possibly related to the intervention. Fatigue, which may have the potential to occur from acupuncture treatment, was naturally and completely resolved after removing the acupuncture needle. There was no noticeable difference observed in the occurrence of adverse events between the two groups (total number of intervention-related adverse events/total number of interventions: 1/69 in the acupuncture group and 0/76 in the sham acupuncture group, *p* = 0.4759).

### Clinical Outcomes Candidates and Sample Size Calculation

**Figure 2:**
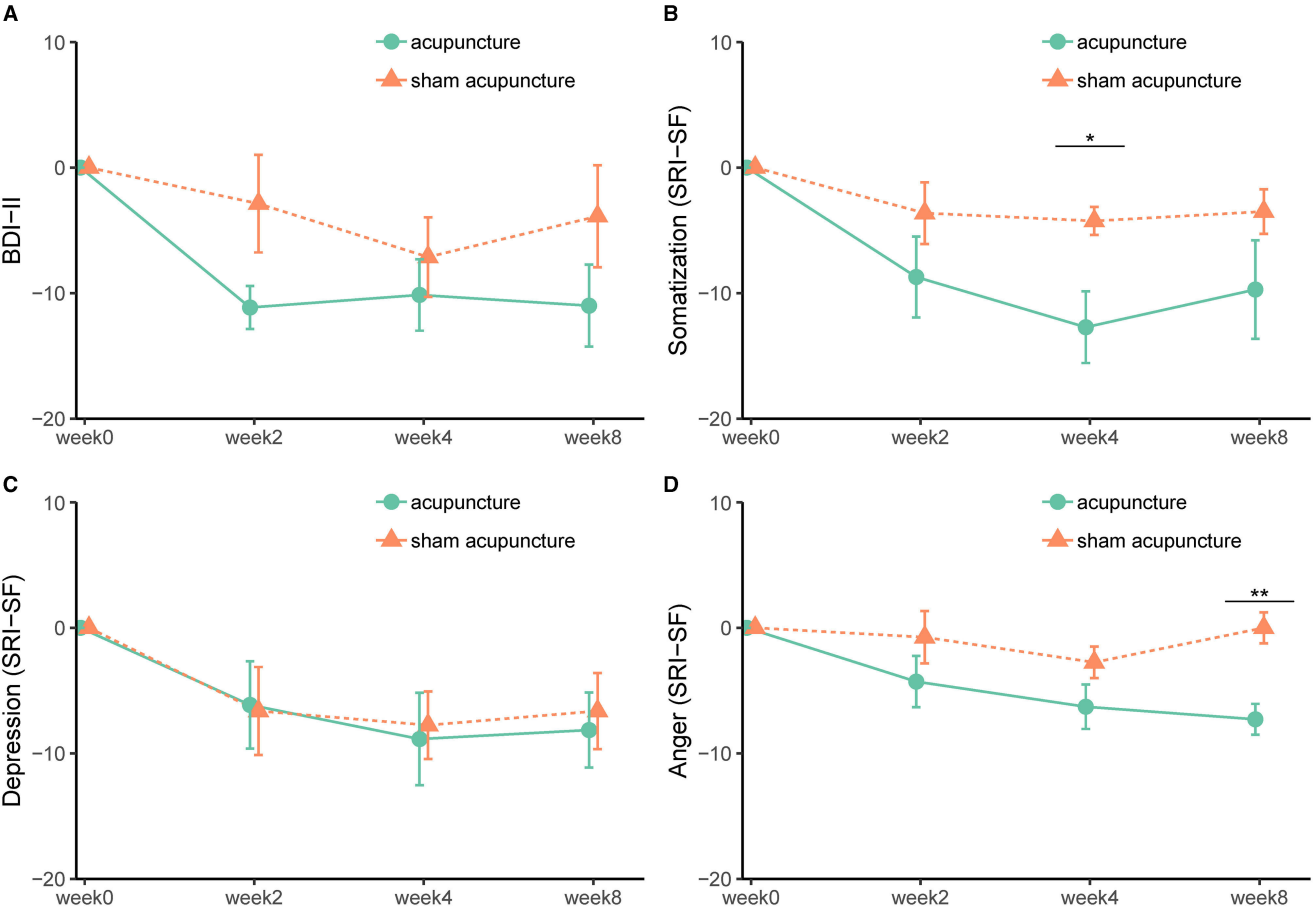
**(A-D)** Effect of acupuncture and sham acupuncture on BDI-II and SRI-SF subscales (somatization, depression, and anger). BDI-II, Beck Depression Inventory-II; SRI-SF, Stress Response Inventory-short form. *, <0.05; **, <0.01.

The mean changes from baseline of clinical outcomes measured at week 0, week 2, week 4, and week 8 are shown in Figure 2, Supplementary Figure 1. The somatization subscale of the SRI-SF seemed to gradually decrease during the treatment sessions in the acupuncture group. The results of repeated measure ANOVA are presented in Supplementary Table 2. As *post-hoc* tests, simple main effects of the group at each time point and results of pairwise comparisons at each time point are presented in Supplementary Tables 3, 4, respectively. There was an interaction between group and time in explaining the VAS of “stuffiness in the chest” [*F*_(3, 39)_ = 3.83, *p* = 0.0170]. Simple main effect of the group was observed at week 2 (*p* = 0.0330). Pairwise comparison showed that the difference at week 2 between the two groups was −39.19 (95% CI −69.81 to −8.55). In the somatization subscale of SRI-SF, the pairwise comparison showed that the difference at week 4 (posttreatment) between the two groups was −8.46 (95% CI −15.55 to −1.38) and the Cohen's *d* effect size was 1.46 (95% CI 0.32–5.50), which referring to large effect size. In the anger subscale of SRI-SF, the pairwise comparison showed that the difference at week 8 (follow-up) between the two groups was −7.29 (95% CI −11.06 to −3.51).

Based on the Cohen's *d* effect size at week 4 (Table 2), the sample size required for a definitive trial can be calculated. If VAS of “stuffiness in the chest” is selected for the primary outcome, the required sample size in each group for a definitive trial with a significance level of 0.05 and power of 0.9 is 33.02. In the case of the somatization subscale of SRI-SF, the required number per group is 10.92.

### Recruitment Rate and Compliance to Intervention

A total of 83.33% (95% CI 60.78–94.16) of screened participants were eligible for the study, based on the inclusion and exclusion criteria. During the 60-week recruiting period, 15 participants were enrolled in the study in one Korean medicine hospital, and the recruitment rate was 1.25 (95% CI 0–2.69) participants per month. Mean compliances for receiving the intervention were 98.57% (95% CI 95.08–100) in the acupuncture group and 95.00% (95% CI 88.68–100) in the sham acupuncture group. All included participants completed the study with a compliance rate of over 80%, and the adherence rate and completion rate were 100% (95% CI 79.61–100) in total participants (Supplementary Table 7).

### Blinding Index

The new blinding index was 0.857 (95% CI 0.598–1.000) in the acupuncture group, indicating that the participants thought that they had received classical acupuncture treatment. In the sham acupuncture group, it was −0.625 (95% CI −1.000 to −0.143), meaning that the participants also thought that they had received classical acupuncture treatment. This is one of the ideal blinding scenarios, therefore, blinding was well-maintained in the sham acupuncture control group throughout the study period (Bang et al., 2004) (Supplementary Table 8).

## Discussion

In this pilot trial, the effect size for acupuncture compared to sham treatment, calculated by Cohen's *d* was >0.2 in all six of the major Hwa-byung symptoms posttreatment. The Cohen's *d* effect size for stuffiness in the chest was 0.81, indicating a large effect size. No serious adverse events were reported in the pilot trial. Based on the results of this pilot trial, we suggest that acupuncture is an acceptable intervention in potential effect and safety for a definitive trial of patients with Hwa-byung.

The feasibility criteria for assessing the success of this pilot trial were predefined as three or more of the major symptoms of Hwa-byung to be improved after the acupuncture treatment compared to sham, with an effect size >0.2 (Lee et al., 2018). Cohen's *d* over 0.2 is considered as small, over 0.5 is considered as moderate, and over 0.8 is considered as large effect size (Cohen, 1988; Prajapati et al., 2010). At posttreatment, the effect size of acupuncture for each of the six Hwa-byung major symptoms compared to sham was over 0.2. The effect sizes for acupuncture for major Hwa-byung symptoms were small to large posttreatment, depending on the particular Hwa-byung symptom.

Of the six major Hwa-byung symptoms, “stuffiness in the chest” was noticeably improved by the acupuncture compared to control in this pilot trial. Stuffiness in the chest is one of the typical somatic symptoms of Hwa-byung, and patients explain that it is caused by suppressed and stagnant anger (Lee et al., 2012b). In a survey of non-pharmacological treatment for Hwa-byung, professional experts in Korean medicine hospitals answered that stuffiness in the chest is the most effective symptom that can be improved by acupuncture treatment (Lee et al., 2009). Acupuncture seems to have the potential to relieve the somatic symptoms of stuffiness in the chest due to psychological problems. Measuring the severity of major Hwa-byung symptoms by VAS was applied in recent clinical trials of Hwa-byung (Lee et al., 2018; Kwak et al., 2019; Choi et al., 2021). However, it is not a validated method for measuring the severity of Hwa-byung symptoms. As validated clinical outcomes, BDI-II and SRI-SF were tested in this pilot study. From the result, participants in the acupuncture group showed better improvement in the somatization subscale of SRI-SF, compared to that in the sham acupuncture group. The somatization subscale of SRI-SF seems to be a good tool for reflecting the effect of acupuncture in patients with Hwa-byung. However, it is still unclear whether it could be the core outcome of patients with Hwa-byung, because there is a lack of study on the core outcome set (Williamson et al., 2012) of Hwa-byung. To determine the primary outcome for a definitive trial, consideration should be given to what is the important outcome for patients with Hwa-byung and whether the tool reflects the improvement by intervention.

Although the expected recruitment rate was four participants per month, the actual recruitment rate in this pilot trial was 1.25 (95% CI 0–2.69) participants per month over 12 months, and this clinical trial ended early, before recruiting 26 participants as planned. The recruitment rate was not consistent for 12 months; seven participants were included in 1 month, and zero in 8 months. In previous acupuncture trials for Hwa-byung, the recruitment rates were reported to be 1.1–2.4 per week (Lee et al., 2007, 2012a; Jung et al., 2008; Choi et al., 2011, 2015), these rates were higher than the results of our pilot trial. When conducting a large-scale trial, recruitment of participants in multiple centers is strongly recommended. Also, in this trial, every participant who was included received treatment with over 80% compliance. More sessions with longer duration were applied in this study, compared to previous studies. Previously, six sessions in 2 weeks (Lee et al., 2007, 2012a; Jung et al., 2008) or four sessions in 2 weeks (Choi et al., 2011, 2015) of acupuncture treatment were tested. Based on the result of this study, 10 sessions of acupuncture treatment over 4 weeks seems to be feasible for patients with Hwa-byung.

As a sham control of acupuncture, minimal acupuncture on nonclassical acupoints was used in this pilot trial. The Bang's blinding index (Bang et al., 2004) was checked to determine whether the blinding procedure requires modification. Most participants in both groups believed that they received classical acupuncture at the posttreatment, and there was no blinding issue raised. However, minimal acupuncture also penetrates the skin-like verum acupuncture. Penetrating the skin appears to be a component that having a physiological activity (Macpherson et al., 2014; Zhang et al., 2016), and as a result, minimal acupuncture does not close to an inert placebo. For the further trial, non-penetrating sham acupuncture methods which are close to an inert placebo or usual care control would be the better options for the control group of acupuncture (Macpherson and Charlesworth, 2020).

This study had several limitations. First, the originally planned sample size was 26 participants (Lee et al., 2018), but the study was completed with 15 participants due to budget and time, despite recruiting for 1 year. Also, the number of this pilot study was not planned to be sufficient for testing hypotheses. This pilot trial was not powered for testing hypotheses about the effect, and the results of the analysis should be interpreted as preliminary. Second, the determination of the primary outcome for a definitive trial still remains uncertain. VAS of major Hwa-byung symptoms has limitations in that it is not a validated tool. The somatization subscale of SRI is a validated tool and appears to be an outcome that can reflect the effect of acupuncture in a patient with Hwa-byung. Further researches on expert consensus and perspective of patients are required to find out whether the somatization subscale of SRI would be the core outcome for patients with Hwa-byung.

In this pilot study, we provided preliminary results on the efficacy and safety of using acupuncture for patients with Hwa-byung for use in a future large-scale clinical trial. The results of this study suggest that acupuncture treatment would be regarded as an acceptable intervention for a full-scale study of psychosomatic symptoms in patients with Hwa-byung. Also, the somatization subscale of SRI is recommended as a tool that seems to measure the effect of acupuncture for patients with Hwa-byung successfully. Large-scale acupuncture clinical trial for Hwa-byung symptoms is recommended to be conducted in multiple centers, together with a feasible participant recruitment plan.

## Data Availability Statement

The original contributions presented in the study are included in the article/Supplementary Material, further inquiries can be directed to the corresponding author/s.

## Ethics Statement

The studies involving human participants were reviewed and approved by The Institutional Review Board of the Daejeon Oriental Hospital of Daejeon University (approval no. djomc-133). The patients/participants provided their written informed consent to participate in this study.

## Author Contributions

J-EK and J-HK: conceptualization. OK: data curation. YC and OK: formal analysis. J-HL and J-HK: funding acquisition and supervision. J-EK, A-RK, H-JP, and J-HK: investigation. YC, I-HP, and J-HK: writing (original draft). YC, I-HP, J-EK, A-RK, J-HL, and J-HK: writing (review and editing). All authors have read and agreed to the published version of the manuscript.

## Funding

This research was supported by grants from Korea Institute of Oriental Medicine (K16122 and KSN1522120).

## Conflict of Interest

The authors declare that the research was conducted in the absence of any commercial or financial relationships that could be construed as a potential conflict of interest.

## Publisher's Note

All claims expressed in this article are solely those of the authors and do not necessarily represent those of their affiliated organizations, or those of the publisher, the editors and the reviewers. Any product that may be evaluated in this article, or claim that may be made by its manufacturer, is not guaranteed or endorsed by the publisher.
