# Supplementary material for: Acupuncture for Psychosomatic Symptoms of Hwa-byung, an Anger Syndrome: A Feasibility Randomized Controlled Trial
Source: Front Psychol. 2021 Sep 24;12:651649. doi: 10.3389/fpsyg.2021.651649 (PMC8498113; doi:10.3389/fpsyg.2021.651649)

Supplementary Material

# Table S1. Adverse events (all causalities) reported during the trial

|  | **Acupuncture group**  **(n=7)** | **Sham acupuncture group**  **(n=8)** |
| --- | --- | --- |
| **Adverse events (Possibly, Probably related)** | | |
| Fatigue | 1 | 0 |
| **Severity of AEs** |  |  |
| Mild | 5 | 2 |
| Moderate | 0 | 3 |
| Severe | 0 | 0 |
| **Causality of AEs** |  |  |
| Definitely related | 0 | 0 |
| Probably related | 0 | 0 |
| Possibly related | 1 | 0 |
| Unlikely related | 0 | 0 |
| Definitely not related | 4 | 5 |
| **Total number of participants with AEs** | 3 | 3 |
| **Total number of AEs** | 5 | 5 |
| **Total number of intervention-related AEs** | 1 | 0 |
| **Total number of interventions** | 69 | 76 |

# Table S2. Results of repeated measure ANOVA in clinical outcomes

|  |  | **df** | ***F*** | **p-value** |
| --- | --- | --- | --- | --- |
| Stuffiness in the chest | group | (1, 13) | 5.56 | 0.0350 |
|  | time | (3, 39) | 13.71 | <0.0001 |
|  | group:time | (3, 39) | 3.83 | 0.0170 |
| Heat-sensation | group | (1, 13) | 0.44 | 0.5210 |
|  | time | (3, 39) | 5.79 | 0.0020 |
|  | group:time | (3, 39) | 0.64 | 0.5910 |
| Pushing-up in the chest | group | (1, 13) | 2.37 | 0.1480 |
|  | time | (3, 39) | 7.37 | 0.0005 |
|  | group:time | (3, 39) | 1.67 | 0.1890 |
| Feeling a mass in the throat | group | (1, 13) | 1.24 | 0.2860 |
|  | time | (1.87, 24.29) | 8.10 | 0.0020 |
|  | group:time | (1.87, 24.29) | 0.81 | 0.4500 |
| Feeling of unfairness | group | (1, 13) | 1.55 | 0.2350 |
|  | time | (1.89, 24.57) | 16.87 | <0.0001 |
|  | group:time | (1.89, 24.57) | 1.08 | 0.3530 |
| Hard feelings or *Hann* | group | (1, 13) | 0.86 | 0.3720 |
|  | time | (1.64, 21.31) | 12.06 | 0.0006 |
|  | group:time | (1.64, 21.31) | 0.95 | 0.3870 |
| BDI-II | group | (1, 13) | 1.94 | 0.1870 |
|  | time | (1.93, 25.11) | 7.92 | 0.0020 |
|  | group:time | (1.93, 25.11) | 1.88 | 0.1740 |
| Somatization (SRI-SF) | group | (1, 13) | 3.88 | 0.0710 |
|  | time | (2.15, 28.01) | 10.04 | 0.0004 |
|  | group:time | (2.15, 28.01) | 2.37 | 0.1080 |
| Depression (SRI-SF) | group | (1, 13) | 0.03 | 0.8620 |
|  | time | (3, 39) | 6.46 | 0.0010 |
|  | group:time | (3, 39) | 0.10 | 0.9600 |
| Anger (SRI-SF) | group | (1, 13) | 6.97 | 0.0200 |
|  | time | (3, 39) | 5.24 | 0.0040 |
|  | group:time | (3, 39) | 3.02 | 0.0410 |

ANOVA: analysis of variance, BDI-II: Beck Depression Inventory-II, SRI-SF: short form of Stress Response Inventory.

# Table S3. Simple main effect of group at each time point by ANOVA in clinical outcomes

|  |  | **df** | ***F*** | **p-value** | **adjusted**  **p-value** |
| --- | --- | --- | --- | --- | --- |
| Stuffiness in the chest | week 2 | (1, 13) | 8.86 | 0.0110 | 0.0330 |
|  | week 4 | (1, 13) | 2.53 | 0.1360 | 0.4080 |
|  | week 8 | (1, 13) | 4.11 | 0.0640 | 0.1920 |
| Heat-sensation | week 2 | (1, 13) | 1.05 | 0.3240 | 0.9720 |
|  | week 4 | (1, 13) | 0.47 | 0.5050 | 0.9999 |
|  | week 8 | (1, 13) | 0.01 | 0.9180 | 0.9999 |
| Pushing-up in the chest | week 2 | (1, 13) | 3.11 | 0.1010 | 0.3030 |
|  | week 4 | (1, 13) | 2.29 | 0.1540 | 0.4620 |
|  | week 8 | (1, 13) | 1.11 | 0.3110 | 0.9330 |
| Feeling a mass in the throat | week 2 | (1, 13) | 1.67 | 0.2190 | 0.6570 |
|  | week 4 | (1, 13) | 1.08 | 0.3180 | 0.9540 |
|  | week 8 | (1, 13) | 0.71 | 0.4160 | 0.9999 |
| Feeling of unfairness | week 2 | (1, 13) | 1.47 | 0.2470 | 0.7410 |
|  | week 4 | (1, 13) | 1.1 | 0.3120 | 0.9360 |
|  | week 8 | (1, 13) | 1.63 | 0.2240 | 0.6720 |
| Hard feelings or *Hann* ^a^ | week 2 | (1, 13) | 2.06 | 0.1750 | 0.5250 |
|  | week 4 | (1, 13) | 0.24 | 0.6290 | 0.9999 |
|  | week 8 | (1, 13) | 0.68 | 0.4250 | 0.9999 |
| BDI-II | week 2 | (1, 13) | 3.43 | 0.0870 | 0.2610 |
|  | week 4 | (1, 13) | 0.49 | 0.4970 | 0.9999 |
|  | week 8 | (1, 13) | 1.79 | 0.2040 | 0.6120 |
| Somatization (SRI-SF) | week 2 | (1, 13) | 1.63 | 0.2250 | 0.6750 |
|  | week 4 | (1, 13) | 8.46 | 0.0120 | 0.0360 |
|  | week 8 | (1, 13) | 2.27 | 0.1560 | 0.4680 |
| Depression (SRI-SF) | week 2 | (1, 13) | 0.01 | 0.9240 | 0.9999 |
|  | week 4 | (1, 13) | 0.06 | 0.8090 | 0.9999 |
|  | week 8 | (1, 13) | 0.13 | 0.7280 | 0.9999 |
| Anger (SRI-SF) | week 2 | (1, 13) | 1.46 | 0.2490 | 0.7470 |
|  | week 4 | (1, 13) | 2.77 | 0.1200 | 0.3600 |
|  | week 8 | (1, 13) | 17.24 | 0.0010 | 0.0030 |

ANOVA: analysis of variance, BDI-II: Beck Depression Inventory-II, SRI-SF: short form of Stress Response Inventory.

# Table S4. Pairwise comparison between groups at each time point in clinical outcomes

|  |  | **acupuncture** | **Sham acupuncture** | **Mean difference**  **(95% CI)** | **Cohen’s d**  **(95% CI)** | **p-value** | **power** |
| --- | --- | --- | --- | --- | --- | --- | --- |
| Stuffiness in the chest | week 2 | -48.43 (31.62) | -9.25 (18.56) | -39.18  (-69.81, -8.55) | 1.51  (0.59, 4.20) | 0.0175 | 0.7701 |
|  | week 4 | -44.29 (35.96) | -21.00 (19.41) | -23.29  (-57.75, 11.18) | 0.81  (-0.40, 2.20) | 0.1610 | 0.3030 |
|  | week 8 | -47.00 (29.89) | -17.75 (26.00) | -29.25  (-60.97, 2.47) | 1.04  (0.14, 3.10) | 0.0676 | 0.4634 |
| Heat-sensation | week 2 | -42.57 (46.93) | -23.00 (25.33) | -19.57  (-64.56, 25.42) | 0.52  (-0.54, 2.47) | 0.3510 | 0.1535 |
|  | week 4 | -32.57 (51.65) | -18.50 (25.26) | -14.07  (-63.11, 34.97) | 0.35  (-1.37, 1.70) | 0.5300 | 0.0952 |
|  | week 8 | -23.86 (48.96) | -21.88 (20.84) | -1.98  (-48.03, 44.07) | 0.05  (-1.61, 1.27) | 0.9230 | 0.0510 |
| Pushing-up in the chest | week 2 | -47.29 (40.90) | -12.50 (35.58) | -34.79  (-78.19, 8.62) | 0.90  (-0.04, 2.61) | 0.1060 | 0.3687 |
|  | week 4 | -46.14 (43.42) | -14.88 (36.73) | -31.27  (-76.92, 14.38) | 0.78  (-0.26, 2.21) | 0.1610 | 0.2856 |
|  | week 8 | -47.57 (36.61) | -25.38 (43.94) | -22.20  (-67.14, 22.75) | 0.55  (-0.46, 2.02) | 0.3050 | 0.1661 |
| Feeling a mass in the throat | week 2 | -23.57 (39.11) | -1.38 (27.21) | -22.20  (-61.22, 16.83) | 0.66  (-0.43, 1.91) | 0.2350 | 0.2187 |
|  | week 4 | -42.71 (45.09) | -23.62 (24.60) | -19.09  (-62.37, 24.19) | 0.53  (-0.70, 2.10) | 0.3440 | 0.1562 |
|  | week 8 | -39.86 (47.10) | -22.38 (33.12) | -17.48  (-64.59, 29.63) | 0.43  (-0.59, 1.93) | 0.4300 | 0.1201 |
| Feeling of unfairness | week 2 | -47.14 (34.85) | -27.62 (27.45) | -19.52  (-55.38, 16.34) | 0.62  (-0.48, 2.24) | 0.2570 | 0.2001 |
|  | week 4 | -49.29 (38.77) | -31.50 (26.39) | -17.79  (-56.30, 20.73) | 0.54  (-0.65, 2.12) | 0.3290 | 0.1607 |
|  | week 8 | -48.29 (37.22) | -27.50 (25.55) | -20.79  (-57.82, 16.25) | 0.65  (-0.38, 2.17) | 0.2410 | 0.2147 |
| Hard feelings or *Hann* | week 2 | -43.71 (39.51) | -20.62 (21.47) | -23.09  (-60.99, 14.81) | 0.73  (-0.41, 2.32) | 0.2010 | 0.2554 |
|  | week 4 | -41.29 (45.05) | -31.88 (27.70) | -9.41  (-53.35, 34.53) | 0.25  (-1.20, 1.70) | 0.6420 | 0.0736 |
|  | week 8 | -43.00 (41.81) | -27.50 (30.94) | -15.50  (-57.81, 26.81) | 0.42  (-0.72, 1.71) | 0.4370 | 0.1175 |
| BDI-II | week 2 | -11.14 (4.53) | -2.88 (10.99) | -8.27  (-17.78, 1.25) | 0.98  (-0.07, 2.46) | 0.0813 | 0.4212 |
|  | week 4 | -10.14 (7.54) | -7.12 (8.97) | -3.02  (-12.23, 6.19) | 0.36  (-0.79, 1.48) | 0.4910 | 0.1001 |
|  | week 8 | -11.00 (8.64) | -3.88 (11.51) | -7.12  (-18.42, 4.17) | 0.70  (-0.21, 2.29) | 0.1960 | 0.2408 |
| Somatization (SRI-SF) | week 2 | -8.71 (8.52) | -3.62 (6.95) | -5.09  (-13.94, 3.77) | 0.65  (-0.43, 2.03) | 0.2340 | 0.2165 |
|  | week 4 | -12.71 (7.54) | -4.25 (3.15) | -8.46  (-15.55, -1.38) | 1.46  (0.32, 5.50) | 0.0250 | 0.7440 |
|  | week 8 | -9.71 (10.40) | -3.50 (5.01) | -6.21  (-16.08, 3.65) | 0.76  (-0.29, 3.31) | 0.1860 | 0.2756 |
| Depression (SRI-SF) | week 2 | -6.14 (9.19) | -6.62 (9.91) | 0.48  (-10.18, 11.15) | -0.05  (-1.18, 1.35) | 0.9240 | 0.0509 |
|  | week 4 | -8.86 (9.72) | -7.75 (7.61) | -1.11  (-11.09, 8.88) | 0.13  (-0.97, 1.28) | 0.8120 | 0.0560 |
|  | week 8 | -8.14 (7.90) | -6.62 (8.57) | -1.52  (-10.71, 7.68) | 0.18  (-1.02, 1.40) | 0.7270 | 0.0626 |
| Anger (SRI-SF) | week 2 | -4.29 (5.41) | -0.75 (5.87) | -3.54  (-9.83, 2.76) | 0.63  (-0.43, 1.95) | 0.2470 | 0.2021 |
|  | week 4 | -6.29 (4.68) | -2.75 (3.54) | -3.54  -8.30, 1.23) | 0.85  (-0.19, 2.26) | 0.1310 | 0.3325 |
|  | week 8 | -7.29 (3.25) | 0.00 (3.50) | -7.29  (-11.06, -3.51) | 2.16  (1.37, 4.44) | 0.0011 | 0.9700 |

Data are shown as mean (SD) or estimate (95% CI)

ANOVA: analysis of variance, BDI-II: Beck Depression Inventory-II, SRI-SF: short form of Stress Response Inventory.

# Table S5. Adjusted mean and sd of two groups at each time point estimated by ANCOVA using baseline values as covariate in clinical outcomes

|  |  | **acupuncture** | **Sham acupuncture** | **Mean difference**  **(95% CI)** | **p-value** | **power** |
| --- | --- | --- | --- | --- | --- | --- |
| Stuffiness in the chest | week 2 | 34.73 (22.16) | 63.23 (22.06) | 28.50 (2.74, 54.26) | 0.0329 | 0.3917 |
|  | week 4 | 36.02 (29.45) | 53.98 (29.32) | 17.97 (-16.27, 52.20) | 0.2752 | 0.1579 |
|  | week 8 | 33.27 (29.02) | 57.26 (28.89) | 23.99 (-9.74, 57.73) | 0.1472 | 0.2585 |
| Heat-sensation | week 2 | 24.98 (25.73) | 43.89 (25.73) | 18.90 (-10.11, 47.92) | 0.1812 | 0.2226 |
|  | week 4 | 34.97 (30.97) | 48.40 (30.97) | 13.43 (-21.50, 48.36) | 0.4185 | 0.1055 |
|  | week 8 | 43.58 (32.86) | 45.11 (32.86) | 1.53 (-35.52, 38.58) | 0.9298 | 0.0506 |
| Pushing-up in the chest | week 2 | 39.43 (30.35) | 57.63 (30.24) | 18.20 (-16.92, 53.32) | 0.2810 | 0.1551 |
|  | week 4 | 38.18 (37.93) | 57.34 (37.79) | 19.17 (-24.72, 63.06) | 0.3601 | 0.1227 |
|  | week 8 | 38.50 (35.53) | 45.31 (35.39) | 6.81 (-34.30, 47.92) | 0.7243 | 0.0597 |
| Feeling a mass in the throat | week 2 | 54.51 (26.83) | 62.05 (26.72) | 7.54 (-23.54, 38.62) | 0.6069 | 0.0712 |
|  | week 4 | 34.20 (32.54) | 40.82 (32.41) | 6.62 (-31.08, 44.33) | 0.7086 | 0.0610 |
|  | week 8 | 37.69 (37.20) | 41.52 (37.05) | 3.83 (-39.28, 46.94) | 0.8497 | 0.0528 |
| Feeling of unfairness | week 2 | 40.59 (31.85) | 57.74 (31.82) | 17.15 (-19.05, 53.36) | 0.3223 | 0.1367 |
|  | week 4 | 39.31 (32.14) | 53.10 (32.10) | 13.80 (-22.73, 50.32) | 0.4266 | 0.1034 |
|  | week 8 | 39.61 (32.05) | 57.72 (32.01) | 18.11 (-18.32, 54.53) | 0.3000 | 0.1462 |
| Hard feelings or *Hann* | week 2 | 43.96 (30.60) | 66.91 (30.60) | 22.94 (-11.56, 57.45) | 0.1730 | 0.2304 |
|  | week 4 | 46.41 (35.84) | 55.64 (35.84) | 9.23 (-31.18, 49.64) | 0.6278 | 0.0687 |
|  | week 8 | 44.65 (37.22) | 60.06 (37.22) | 15.41 (-26.57, 57.38) | 0.4394 | 0.1003 |
| BDI-II | week 2 | 37.63 (7.15) | 46.20 (7.15) | 8.57 (0.51, 16.63) | 0.0390 | 0.5206 |
|  | week 4 | 38.64 (7.12) | 41.94 (7.12) | 3.29 (-4.74, 11.32) | 0.3892 | 0.1137 |
|  | week 8 | 37.72 (7.77) | 45.25 (7.77) | 7.53 (-1.24, 16.30) | 0.0858 | 0.3606 |
| Somatization (SRI-SF) | week 2 | 13.12 (6.57) | 17.39 (6.57) | 4.27 (-3.15, 11.70) | 0.2339 | 0.1818 |
|  | week 4 | 9.08 (4.09) | 16.80 (4.09) | 7.72 (3.10, 12.34) | 0.0034 | 0.9018 |
|  | week 8 | 12.17 (6.50) | 17.47 (6.50) | 5.30 (-2.04, 12.65) | 0.1418 | 0.2652 |
| Depression (SRI-SF) | week 2 | 12.51 (7.16) | 15.18 (7.15) | 2.66 (-5.53, 10.85) | 0.4923 | 0.0889 |
|  | week 4 | 9.88 (6.07) | 13.98 (6.05) | 4.11 (-2.83, 11.04) | 0.2212 | 0.1904 |
|  | week 8 | 11.09 (7.37) | 14.67 (7.36) | 3.58 (-4.85, 12.01) | 0.3730 | 0.1185 |
| Anger (SRI-SF) | week 2 | 10.58 (4.69) | 11.00 (4.66) | 0.42 (-5.09, 5.93) | 0.8714 | 0.0520 |
|  | week 4 | 7.90 (3.80) | 9.59 (3.78) | 1.69 (-2.77, 6.16) | 0.4249 | 0.1039 |
|  | week 8 | 6.53 (3.40) | 12.66 (3.38) | 6.14 (2.14, 10.13) | 0.0058 | 0.8450 |

Data are shown as mean (SD) or estimate (95% CI)

ANCOVA: analysis of covariance, BDI-II: Beck Depression Inventory-II, SRI-SF: short form of Stress Response Inventory.

# Table S6. Number of participants showed reliable improvement at week 4 using reliable change index (RCI)^a^

|  | **Acupuncture** | | **Sham acupuncture** | |
| --- | --- | --- | --- | --- |
|  | **No change** | **Improved** | **No change** | **Improved** |
| Stuffiness in the chest | 4 | 3 | 6 | 2 |
| Heat-sensation | 4 | 3 | 8 | 0 |
| Pushing-up in the chest | 4 | 3 | 7 | 1 |
| Feeling a mass in the throat | 4 | 3 | 7 | 1 |
| Feeling of unfairness | 3 | 4 | 5 | 3 |
| Hard feelings or *Hann* ^a^ | 4 | 3 | 4 | 4 |
| BDI-II | 3 | 4 | 6 | 2 |
| Somatization | 5 | 2 | 6 | 2 |
| Depression | 3 | 4 | 8 | 0 |
| Anger | 5 | 2 | 8 | 0 |

^a^ Reliable change index (RCI) was calculated using the formula of discrepancy between the week 4 and week 0 divided by standard error of the difference. Participants whose RCI is higher than 1.645 were classified as “improved”.

BDI-II: Beck Depression Inventory-II, SRI-SF: short form of Stress Response Inventory.

Reference

Duff, K. 2012. 'Evidence-based indicators of neuropsychological change in the individual patient: relevant concepts and methods', *Arch Clin Neuropsychol*, 27: 248-61.

# Table S6. Recruitment rate and compliance to interventions

| **Feasibility outcomes** | **Total**  **(n=15)** | **Acupuncture**  **(n=7)** | **Sham acupuncture**  **(n=8)** |
| --- | --- | --- | --- |
| Screened participants | 18 | - | - |
| Eligible participants | 15 | - | - |
| Months of recruitment | 12 | - | - |
| Participants whose compliance higher than 80 % | 15 | 7 | 8 |
| Participants who completed the study | 15 | 7 | 8 |
| Recruitment rate (95% CI), participant/month | 1.25 (0, 2.69) | - | - |
| Mean compliance to interventions (95% CI), % | 96.67 (93.25, 100) | 98.57 (95.08, 100) | 95.00 (88.68, 100) |
| Adherence rate (95% CI), % | 100 (79.61, 100) | 100 (64.57, 100) | 100 (67.56, 100) |
| Completion rate (95% CI), % | 100 (79.61, 100) | 100 (64.57, 100) | 100 (67.56, 100) |

# Table S7. Blinding test

|  | **Acupuncture group** | **Sham acupuncture group** | **p-value** |
| --- | --- | --- | --- |
| Classical acupuncture | 6 (85.71 %) | 6 (75.00 %) | 0.999 |
| Non-classical acupuncture | 0 (0.00 %) | 1 (12.50 %) |  |
| Don’t know | 1 (14.29 %) | 1 (12.50 %) |  |
| New Blinding Index (95% CI) | 0.857 (0.598, 1.000) | -0.625 (-1.000, -0.143) |  |

**Figure S1. Effect of acupuncture and sham acupuncture on major symptoms of Hwa-byung measured by Visual Analog Scale (VAS)**


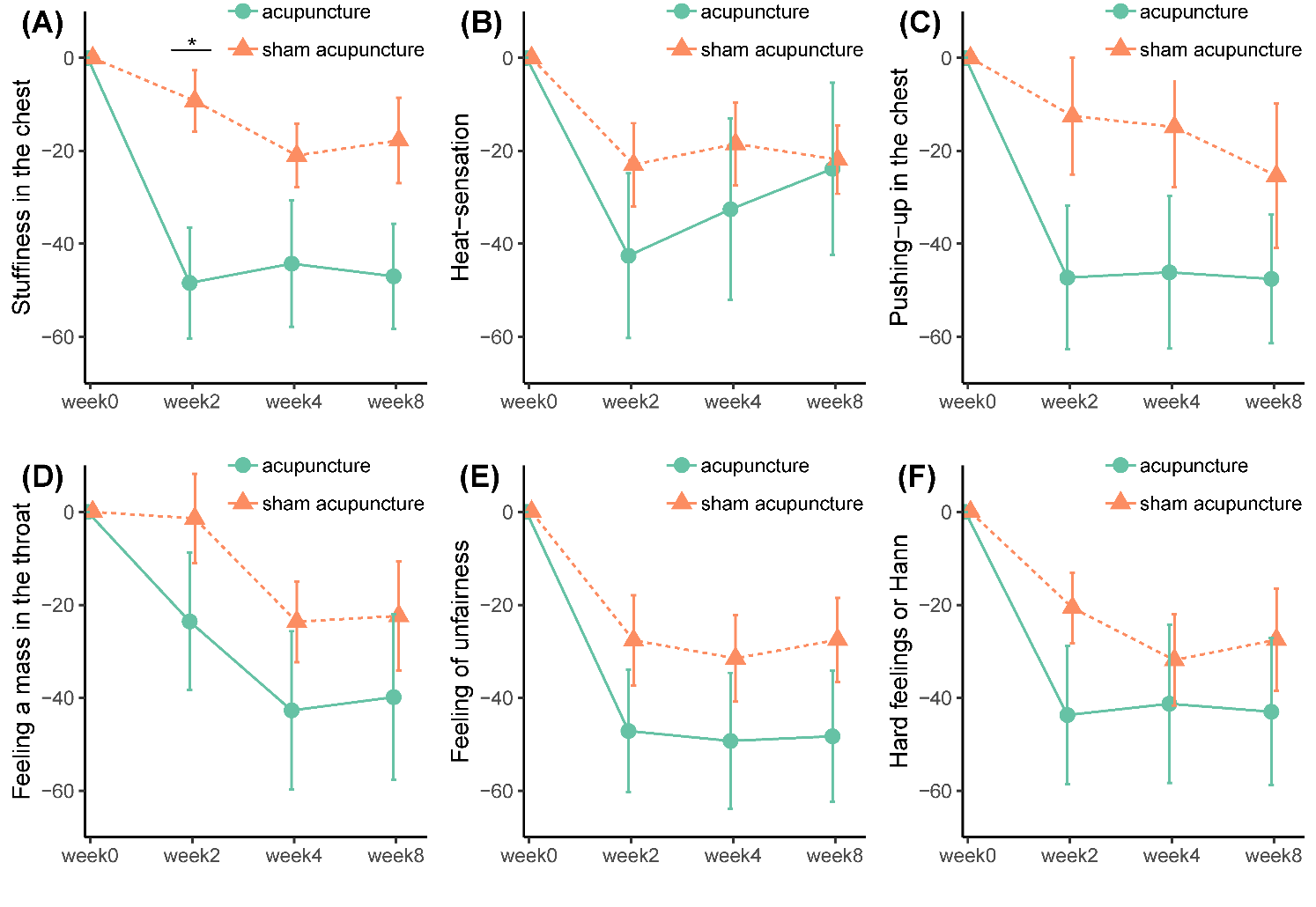

Supplement: Supplementary file 1 [file Data_Sheet_1.docx]
